# Preparation and Performance Evaluation of Temperature-Resistant and Salt-Resistant Gels

**DOI:** 10.3390/gels10050337

**Published:** 2024-05-16

**Authors:** Xudong Li, Meilong Fu, Jiani Hu

**Affiliations:** Hubei Provincial Key Laboratory of Oil and Gas Drilling and Production Engineering, School of Petroleum Engineering, Yangtze University, Wuhan 430100, China; 201801257@yangtzeu.edu.cn (X.L.); hujiani0125@163.com (J.H.)

**Keywords:** high-temperature and high-salt reservoir plugging, polymer gel, plugging rate, performance evaluation

## Abstract

In order to improve the plugging performance of high-temperature and high-salt oil reservoir plugging agents, this paper utilizes a copolymer composed of acrylamide and 2-acrylamide-2-methylpropanesulfonic acid (AM/AMPS) as the polymer, polyethyleneimine as the cross-linking agent, and nylon fiber as the stabilizer to develop a high-temperature- and high-salt-resistant gel system. This study analyzed and evaluated the temperature resistance, salt resistance and blocking performance of the gel system. The evaluation results show that the gel-forming strength of this gel system can reach an H level, and it has good thermal stability at the high temperature of 130 °C. At the high salinity of 240,720 mg/L, the syneresis rate remains below 2.5%, and the gel-forming time is greater than 15 h; the higher the temperature, the shorter the gelling time. The results of our sand-filled pipe-plugging experiment show that the gel system can adapt to sand-filled pipes with different levels of permeability, and reaching a plugging rate of 94%.

## 1. Introduction

Currently, there is a growing emphasis on the exploration and extraction of global petroleum reserves, particularly in high-temperature and high-salt reservoirs. However, these reservoirs are prone to developing cracks and high-permeability channels in the mining process, resulting in increased water production [1]. The heterogeneity of high-temperature and high-salinity reservoirs is pronounced. Therefore, during the process of water injection development, the reservoir environment deteriorates further, making it easier for injected water to be channeled along a high-permeability layer, resulting in premature water production in oil wells [2].

Research has demonstrated that polymer gel systems are promising solutions for addressing the issue of water production issues in oil reservoirs characterized by high temperatures and high concentrations of salt [3,4,5]. A polymer system comprises polymers, cross-linkers, and various auxiliary reagents. The polymer system is injected into the formation water channel, where it undergoes a cross-linking reaction and transforms into a gel after aging at a specific temperature for a defined period. The gel effectively blocks the water outlet channel, leading to a reduction in water outflow rates and an improvement in the recovery rates. Polymer gels designed for water plugging in high-temperature and high-salinity oil reservoirs must exhibit exceptional resistance to heat and salt, and long-term stability. Currently, research and development efforts in temperature-resistant and salt-resistant polymer gel systems primarily focus on two categories: in situ cross-linked polymer gels and pre-cross-linked gels [6,7,8]. In the in situ cross-linked polymer gel approach, polymers (or monomers), cross-linking agents, and additives are injected into the target formation, resulting in the formation of a gel with a three-dimensional network structure through cross-linking reactions [9]. On the other hand, the pre-cross-linked polymer gel method involves creating a pre-cross-linked gel on the surface by combining polymers, cross-linking agents, and additives. This gel is then injected into the target formation in particle form. The particles undergo expansion and aggregation and occupy the surrounding environment, effectively blocking the formation responsible for water production [10].

Zhang et al. [11] utilized gel particles in conjunction with the combination of partially hydrolytic polyacrylamide (HPAM)/Cr^3+^. This allowed the gel particles to evenly and densely cover fracture channels, resulting in a significant enhancement of crack sealing efficiency. Their study showed that the HPAM/Cr^3+^ system has the ability to completely obstruct the pore channel, resulting in a 10.5% increase in the recovery rate. Li et al. [12] created a gel system with high temperature and high strength properties. The gel is formed through covalent bonds and consists of a main polymer (1.5% HPAM), a cross-linking agent (0.8% hexamethylenetetramine + 1.6% Methyl p-hydroxybenzene), a temperature-resistant polymer (0.4%), a toughening agent (1.2% fiber), and a gel control agent (0.3% oxalic acid). The gel is capable of withstanding temperatures up to 150 °C. The test findings indicate that the gel solution demonstrates favorable rheological properties at normal room temperature and can undergo cross-linking to form a gel at elevated temperatures. The plugging test demonstrated that the gel is capable of sealing a 5 mm crack under a pressure gradient of up to 0.25 MPa/cm. Furthermore, the gel’s strength remains consistently stable for a duration of one month. The gel exhibits resistance to the metal cations present in drilling fluids and the elevated ion concentrations found in formation water, including Na^+^, K^+^, Mg^2+^, and Ca^2+^. Once the gel breaker is introduced, it will effectively break down the gel, resulting in the contamination of the reservoir by the gel breaker products. Bai et al. [13] created a gel system by dispersing low-molecular-weight polymers with nanoparticles. The rheology and gel characteristic test results indicate that the gelling agent exhibits excellent shear resistance. The gel maintains stability even when subjected to extreme shear rates. The experimental results demonstrate that the adhesive can uniformly expand within the crevices and, upon gel formation, effectively occupy the entire crack void, thereby enhancing its water-blocking capabilities. In the case of multi-level cracks, the gel exhibits selective filling properties, primarily occupying spaces with higher concentrations of water. Liu et al. [14] created a gel system using a terpolymer and a novel cross-linking technique. They investigated its performance in extremely high-temperature environments. The research findings indicate that the gel system is capable of establishing a durable network structure throughout the temperature range of 120 °C to 200 °C, and it exhibits commendable thermal stability over an extended period of time. The gel system retains a significant portion of its original viscosity and viscoelasticity even after being subjected to mechanical shear or shear in porous media. The findings of the plugging experiment demonstrated that the plugging rate of the 10 sand-filled pipes exceeded 97%, suggesting excellent plugging performance. Jiang et al. [15] created a composite hydrogel with the ability to significantly decrease the rate of leakage. This hydrogel mostly consisted of polyvinyl alcohol, borax, sodium silicate, and other substances. It had excellent gel strength even at a temperature of 60 °C and demonstrated long-term stability in performance when exposed to simulated geological water conditions with a salinity of 12,500 mg/L. The fundamental characteristics of the hydrogel remained unaltered when submerged in water or gasoline. The hydrogel exhibited excellent load-bearing capacity, as demonstrated by indoor plug simulation testing. Huang et al. [16] created a gel plugging agent with a low initial viscosity. The agent was made up of an LH polymer, CYJL cross-linking agent, citric acid at a concentration of mg/L, sodium sulfite, and sodium polyphosphate. This study adjusted the composition of the polymer, the dosage of cross-linking agents, and other additives. The gel’s initial test viscosity was below 10 mPa·s. The core flooding experiment showed that when the permeability of the core was 3.9 μm^2^, more than 99% of the flow was blocked. The gel system exhibited excellent plugging capabilities. The injection of a 0.1 PV gel to seal the core resulted in a 13.6% increase in the recovery factor. Lv et al. [17] utilized a self-formulated nanographite polymer and phenolic resin as a cross-linking agent to fabricate a gel system that exhibits high resistance to both temperature and salt. According to their investigation, the gel system demonstrated a high temperature resistance of 150 °C and maintained stability in a salinity of 300,000 mg/L. The gel system’s microstructure was examined using differential scanning calorimetry. The findings demonstrated that the altered nanographite enhanced the compactness of the gel network structure and effectively enhanced its resistance to high temperatures and salt. Kang et al. [18] utilized free radical polymerization to synthesize a novel variety of pre-cross-linked gel particles. The polymerization process involved the utilization of acrylic acid and acrylamide as monomers that undergo polymerization. N, N-methylene bisacrylamide is used as a cross-linking agent, while ammonium persulfate acts as an initiator. Additionally, sawdust particles are incorporated as a reinforcing agent. This work investigated the microstructure, swelling behavior, viscoelastic properties, yield strength, creep recovery behavior, and shear resistance of the pre-cross-linked gel particle in formation water. The gel particles that had been pre-cross-linked were subjected to core flooding tests. An investigation was conducted to evaluate the gel’s blocking performance. The experimental results indicated that the pre-cross-linked gel (1:5) had a swelling ratio of 8 in formation water and had favorable viscoelasticity, shear resistance, and a greater yield stress. The plugging rate of this pre-cross-linked gel was exceptionally high, reaching 91.9%. Ran et al. [19] utilized a copolymer consisting of acrylamide and 2-acrylamido-2-methylpropanesulfonic acid, along with a modified water-soluble phenolic resin, to synthesize a gel that exhibited prolonged durability in aqueous environments. A comparative analysis between a water-soluble phenolic resin and a phenolic composite cross-linking agent revealed that the use of water-soluble phenolic resin can lead to cost reduction and enhance the strength of the gel system. Zhi et al. [20] developed a weak gel system by cross-linking a hydrophobic association polymer with a cross-linking agent based on phenolic resin. After 90 days of aging, the average viscosity retention rate of this system exceeded 80%. The gel’s network structure could remain intact for a period ranging from 5 to 90 days. This study evaluated the efficacy of the weak gel by conducting core flow experiments to assess its blocking ability. The experimental findings demonstrated that at three distinct temperatures, namely 5, 40, and 200 °C, the rate of plugging exceeded 85%, indicating a commendable plugging performance. Sun et al. [21] added calcite particles to a standard pre-cross-linked gel system and conducted extensive tests to assess the effectiveness of its plugging performance under different calcite filling circumstances. The experimental results suggest a direct relationship between the size and concentration of calcite particles and the PPG breakthrough pressure gradient. Moreover, the plugging rate becomes more efficient as the size and concentration of calcite particles rise. This work provides new and valuable information on how to treat pre-cross-linked gel systems in order to effectively manage water in fractured reservoirs. However, the majority of gel systems developed by previous researchers exhibit low gelation temperatures and lack stability in highly mineralized environments, making them more suitable for reservoirs with medium-to-low temperatures. Gel systems designed for high-temperature and high-salinity reservoirs have only been observed for approximately 30 days, indicating a relatively short research duration. Therefore, the development of a water-plugging gel that is resistant to high temperatures and high salinity, and possesses long-term stability, remains necessary.

This study introduces the development of a gel system that is both resistant to high temperatures and tolerant of high salt concentrations. The gel system exhibited stability at a temperature of 130 °C and a salinity of 24 × 10^4^ mg/L. The selected polymer for this study is AM/AMPS, which exhibits a specific cross-linking characteristic in the AM group. The gel’s stability can be improved and its susceptibility to damage reduced by the formation of a network structure through cross-linking with a cross-linking agent. The AMPS group exhibits a specific water absorption capacity, enabling it to establish hydrogen bonds or ionic interactions with water molecules, regulating water balance within the gel system and preventing excessive syneresis. The chosen cross-linking agent, polyethyleneimine, offers a higher number of cross-linking points, leading to increased cross-linking between polymer chains and enhanced network structure stability. Nylon fiber serves as the stabilizer, acting as a reinforcing agent in the gel system to improve structural integrity and inhibit gel dispersion. The optimal system concentration was determined by adjusting the dosage of each component. Additionally, this study examined the impact of temperature on gelling time; conducted experimental assessments on the strength, temperature resistance, salt resistance, and blocking performance of the gel system; and elucidated the experimental findings based on the cross-linking mechanism.

## 2. Results and Discussion

### 2.1. Synthesis of a Novel Heat- and Salt-Resistant Gel Plugging Agent

#### 2.1.1. Optimization of Polymer Dosage

**Figure 1:**
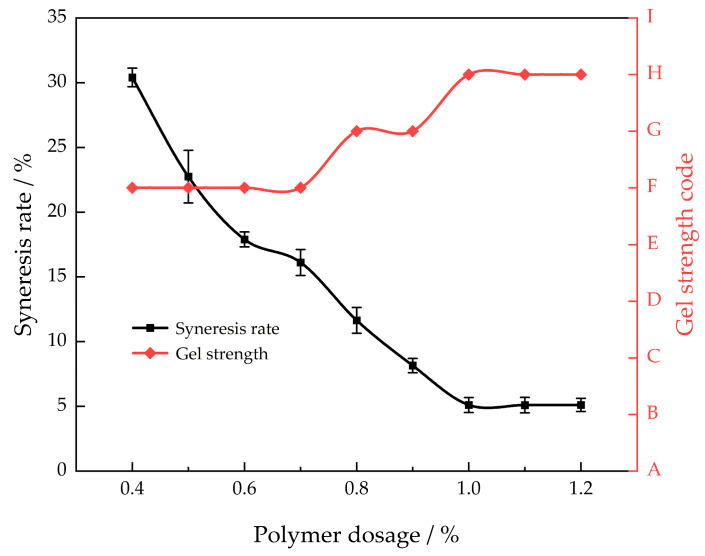
Different polymer concentrations’ gel strength and syneresis rate after 30 days of aging at 130 °C.

This article chose AM/AMPS as the main gel agent. In comparison to the conventional HPAM gel system, the polymerization of acrylamide and AMPS monomers effectively inhibits the hydrolysis of the amide group, thereby enhancing the polymer’s temperature and salt resistance [22]. Thus, various mass fractions of AM/AMPS were selected, in combination with a constant mass fraction of 0.1% polyethyleneimine, for polymer dosage preference experiments conducted at a temperature of 130 °C. The aim was to investigate the impact of different mass fractions of AM/AMPS on the rate of gel syneresis over a period of 30 days (Figure 1) in order to determine the most effective polymer dosage.

Figure 1 illustrates that as the mass percentage of AM/AMPS increases, the syneresis rate of the gel system progressively decreases. Upon reaching a mass fraction of 1.0% for AM/AMPS, the rate of syneresis stabilizes. The presence of the methylpropylsulfonic acid group in AMPS enhances the hydrophilicity of the gel system, preventing gel contraction and enhancing the system’s resistance to temperature variations [23]. With an increase in polymer concentration, gel strength initially rises from the F level and then reaches a stable level at H. By augmenting the mass fraction of the polymer, the number of groups involved in the cross-linking process is likewise enhanced, thereby intensifying the connection between the primary agent and the cross-linking reaction. The network structure resulting from the cross-linking reaction becomes denser and more rigid as the degree of cross-linking of the linking agent increases. Hence, the most effective concentration of the polymer is 1.0%.

#### 2.1.2. Optimization of Cross-Linking Agent Dosage

**Figure 2:**
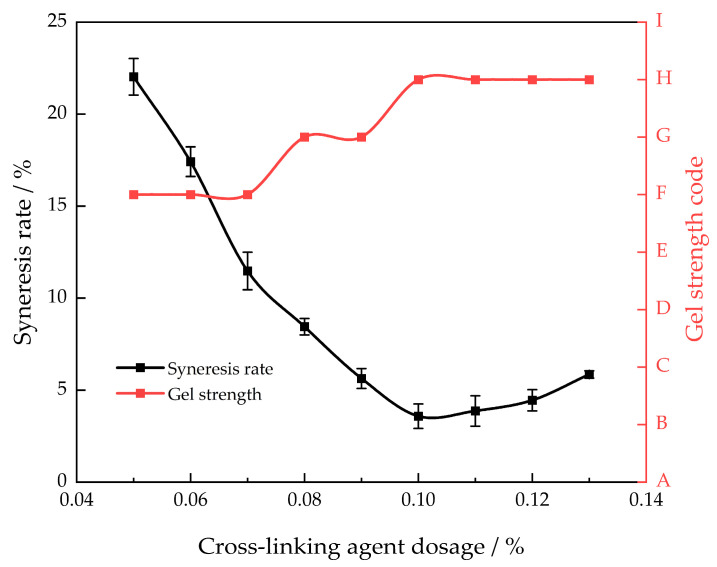
Different cross-linking agent concentrations’ gel strength and syneresis rate after 30 days of aging at 130 °C.

This article used polyethyleneimine as the gel cross-linking agent [24]. This cross-linking agent is a highly reactive substance that is ecologically benign and has low toxicity. Polymers can be easily cross-linked to create a high-strength gel, enhancing their performance in high-temperature conditions. A polymer with a mass fraction of 1.0% was chosen to investigate the impact of cross-linker dosage on the strength of the gel system and the rate of syneresis (30 days) at a temperature of 130 °C (Figure 2).

As the proportion of polyethyleneimine increases, the syneresis rate of the gel system exhibits a pattern of initially decreasing and the increasing. At a polyethyleneimine mass fraction of 0.1%, the syneresis rate decreases to its minimum, while the degree of gel cross-linking reaches its maximum. When the mass fraction of polyethyleneimine is below 0.1%, the syneresis rate exceeds 5%, suggesting inadequate cross-linking. The syneresis phenomenon is evident because of the higher average molecular weight between the cross-linking locations in the gel system and the relatively small effective cross-linking portion. When the mass fraction of polyethyleneimine surpasses 0.1%, the rate at which the gel system contracts due to syneresis increases. This suggests that the level of cross-linking is excessively high, resulting in a decrease in the length of the effectively cross-linked segments and a reduction in the volume of the cross-linked network structure. Consequently, the water absorption rate of the entire gel system decreases, leading to an increase in the syneresis rate [25]. By increasing the mass fraction of the cross-linking agent, the strength of the gel system improved from an initial F level to a stable H level. Hence, the most effective concentration of the cross-linking agent was 0.1%.

#### 2.1.3. Optimization of Stabilizer Dosage

**Figure 3:**
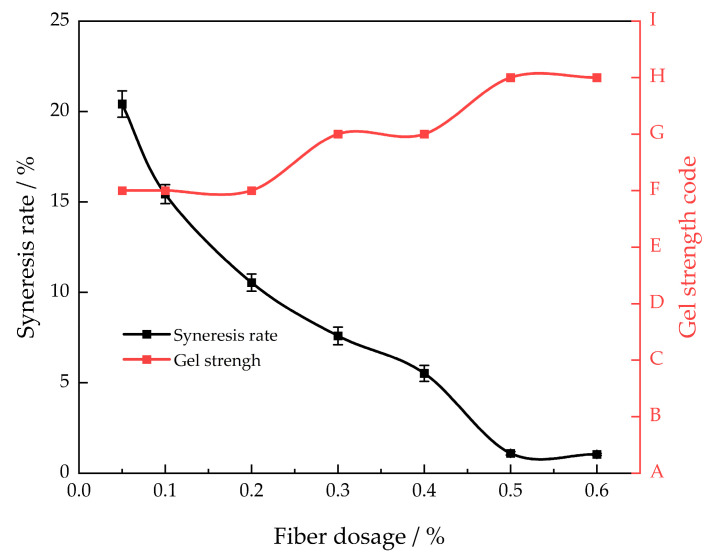
Different nylon fiber concentrations’ gel strength and syneresis rate after 30 days of aging at 130 °C.

Nylon fiber was used as the gel stabilizer in this article. This stabilizer can be uniformly distributed in an adhesive solution to significantly enhance the stability and strength of the gel [15]. The mass fraction of the polymer was 1.0% and the mass fraction of the cross-linking agent was 0.1%, the gelling temperature was 130 °C, and the influence of different amounts of nylon fiber on the gel syneresis rate (30 days) was examined (Figure 3).

As the proportion of the stabilizer grows, the rate at which the gel system releases liquid decreases initially and eventually reaches a stable level. At a mass fraction of 0.5%, the stabilizer causes the gel system’s syneresis rate to decrease to its lowest point. We continued to gradually raise the amount of stabilizer used, as the rate of syneresis remained consistently below 5%. The gel system’s strength gradually rises from level F to level H as the mass fraction of the stabilizer increases. At a mass fraction of 0.5%, the stabilizer achieves its maximum gel strength, denoted as level H. Hence, the most effective amount of stabilizer is 0.5%.

**Figure 4:**
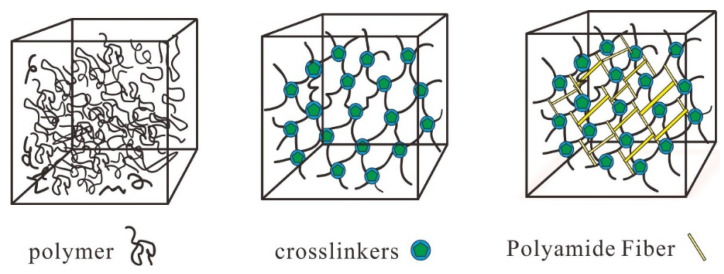
Schematic diagram of the structural strength of the PA fiber-reinforced gel system.

The stabilizer functions to enhance the strength of the gel network structure. Upon injection into the formation, the gel solution enhances the polymer network structure by interconnecting with the polymer molecules, hence augmenting the strength of the gel system (Figure 4).

#### 2.1.4. Determination of Gelling Time

**Figure 5:**
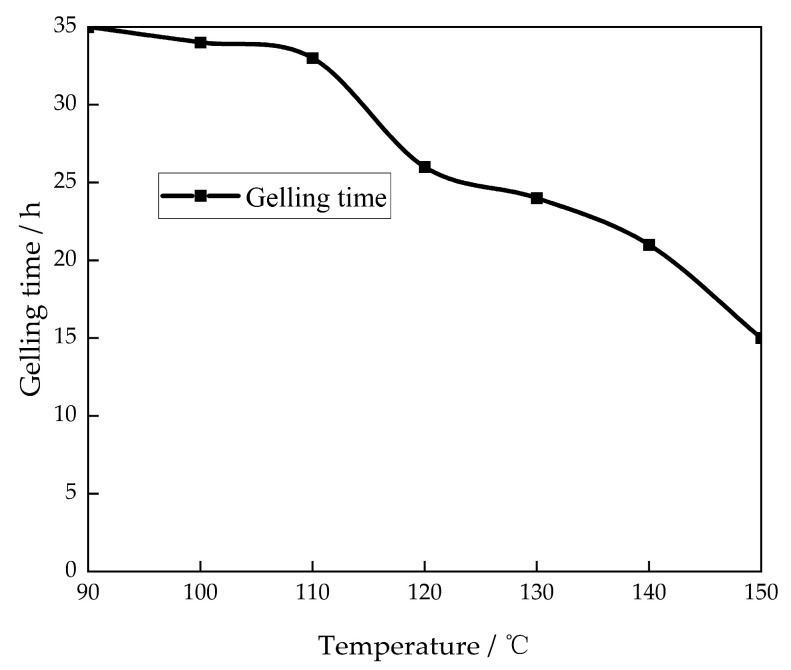
Effect of temperature on the gelling time.

The prepared gel solutions were placed at temperatures of 90 °C to 150 °C to examine the effect of temperature on the gelation time (Figure 5).

The gelling time of the gel system decreases as the temperature increases. At a temperature of 150 °C, the gelation time is a minimum of 15 h. The primary factor is that as the temperature rises, the mobility of free radicals in the adhesive solution accelerates, leading to more profound cross-linking between the polymer and the cross-linking agent. Consequently, the formation of the gel network structure occurs at a faster rate, resulting in the gel system. As the temperature increases, the gelling time decreases [26].

### 2.2. Performance Evaluation of a Novel Heat- and Salt-Resistant Gel Plugging Agent

#### 2.2.1. Temperature Resistance Performance Evaluation

**Figure 6:**
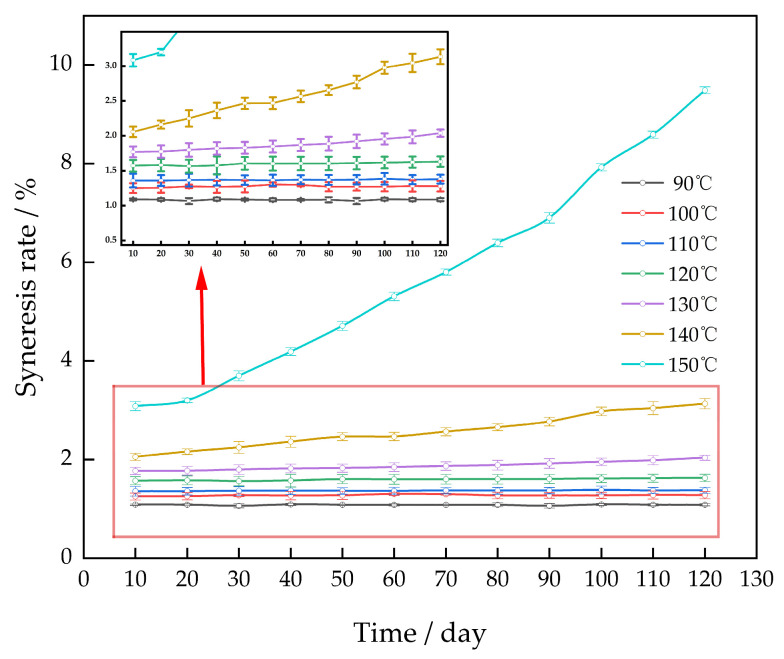
Effect of temperature on the gel syneresis rate.

The gel system was subjected to aging at temperatures of 90 °C to 150 °C for a duration of 120 days in order to investigate the impact of ambient temperature on the rate of gel syneresis (Figure 6).

The gel system had a syneresis rate of less than 3% after 120 days when the ambient temperature was below 140 °C. It was discovered that the syneresis rate of the gel system remained nearly constant as the number of days rose, as long as the temperature did not exceed 130 °C. The primary factor was the excellent temperature tolerance of the cross-linking agent polyethyleneimine, which, in turn, enabled the polymer to efficiently suppress hydrolysis. Hence, when the cross-linking agent undergoes cross-linking with the polymer, it results in the formation of a three-dimensional network structure that exhibits exceptional resistance to high temperatures.

#### 2.2.2. Salt Tolerance Performance Evaluation

**Figure 7:**
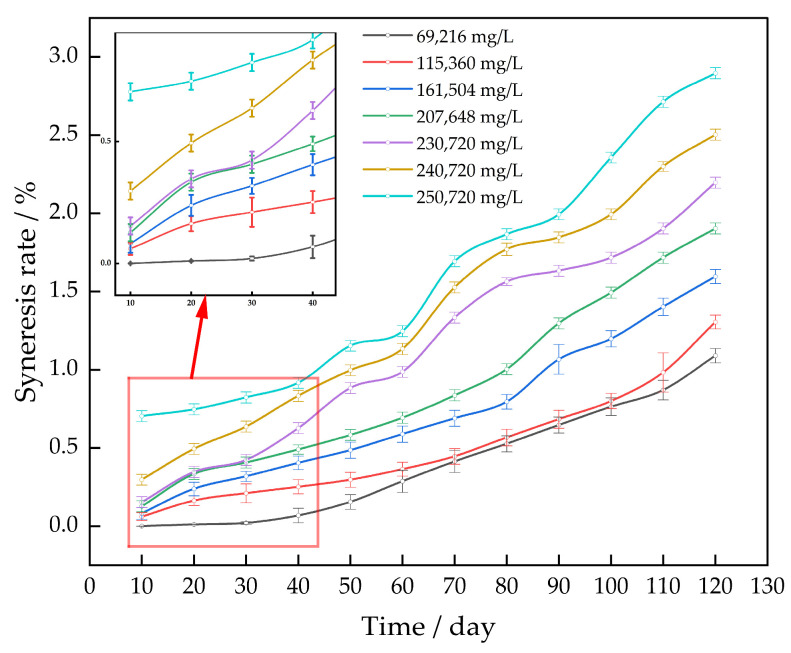
Effect of salinity on the gel syneresis rate.

The gel systems were immersed in simulated formation water with varying salt levels at 130 °C and aged for 120 days. This was carried out to investigate the impact of formation water salinity on the pace at which the gel system undergoes syneresis. The salinity of the formation water at the oil field site is 230,720 mg/L. This article involved diluting simulated formation water with varying amounts of distilled water: 30%, 50%, 70%, and 90%. Additionally, high-concentration simulated formation water was generated using a 3% NaCl solution (Figure 7).

As the concentration of salt in the simulated formation water increases, the rate of separation in the gel system increases with longer periods of age. The gel syneresis rate of the simulated bottom water, with a salinity of 250,720 mg/L, is less than 3%. The inclusion of the sulfonic acid group in the AM/AMPS copolymer effectively inhibits the complexation reaction between the AM group and the cation. Complexation reduces the hydrophilicity of the gel and causes syneresis. Thus, if the complexation reaction is inhibited, it will also hinder the contraction of the gel system.

#### 2.2.3. Plugging Performance Evaluation

The plugging experiment utilized a gel system consisting of 1% AM/AMPS, 0.1% polyethyleneimine, and 0.5% nylon fiber. For this experiment, five types of sand-filled tubes with varying permeability were utilized and labeled as No. 1–5, correspondingly. The gel solution was injected into five sand-filled tubes with distinct levels of permeability and subjected to a temperature of 130 °C to induce gelation. Subsequently, it was displaced by injection into simulated formation water. The rate of injection was 0.5 mL/min and the volume of injection was 0.5 PV. Table 1 displays the test findings, and Table 2 presents the physical specifications of the sand-filled pipeline.

Table 1 demonstrates that the injection of gel into the sand-filled pipes significantly decreased the permeability of the water phase and achieved a plugging rate of 90%. This indicates that the gel system has excellent plugging performance. During the plugging process, adhesive was introduced into the sand-filled pipe and began to occupy the narrow passages between the sand particles. A cross-linking reaction took place at a specific temperature, resulting in the formation of a blocking layer at the pore mouths. The gel forms a network structure that binds water, resulting in increased resistance to seepage and providing a blocking effect. As the sand-filled pipe becomes more permeable, the pressure gradient required for breakthrough reduces steadily. However, the rate at which the pipe becomes clogged remained relatively constant. This suggests that the gel system was effective in plugging the pipe regardless of its permeability, with higher permeability resulting in better plugging effects. A greater value corresponds to a more effective blocking effect.

#### 2.2.4. Gel microstructure Characterization

**Figure 8:**
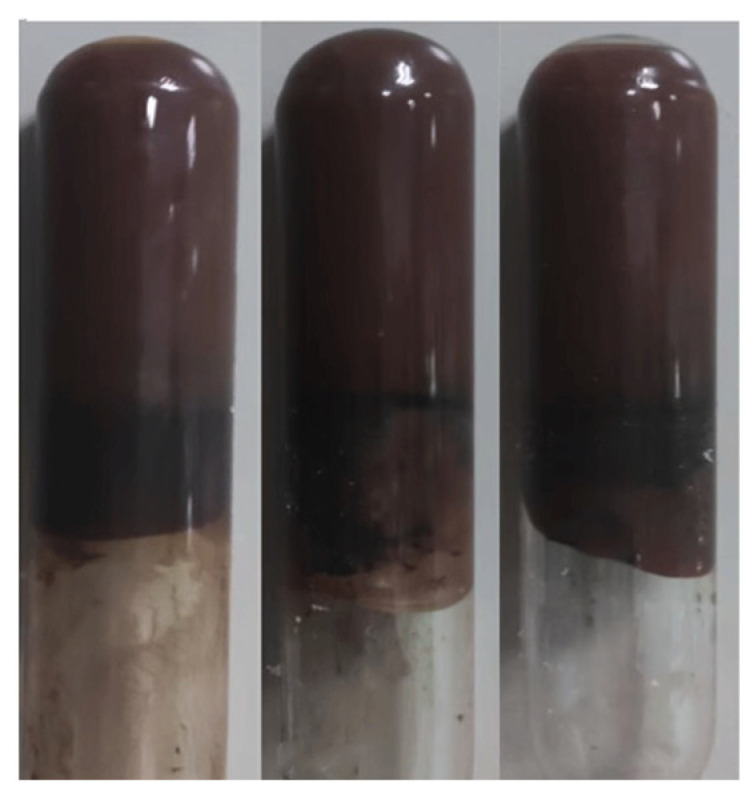
The form of the glue after gelatinization.

Upon undergoing a cross-linking reaction at elevated temperatures, the glue liquid transformed into a gel, exhibiting a dark brown color and possessing a strength rating of H, as depicted in Figure 8.

**Figure 9:**
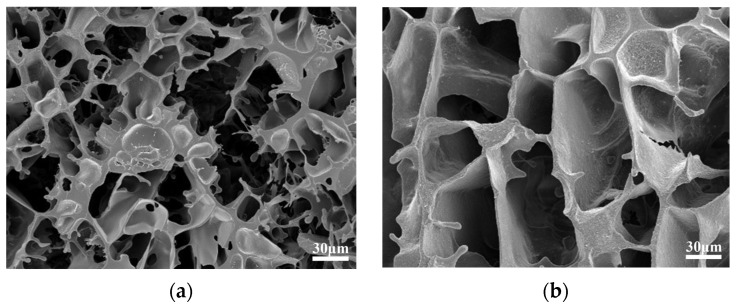
Microscopic morphological characterization of gel. (**a**) gel structure after aging at 130 °C; (**b**) gel structure after aging at 130 °C.

Scanning electron microscopy (SEM) was used to observe the internal structure of the gel system [27]. The gel sample was placed on newly cleaved mica sheets and quickly frozen in liquid nitrogen. The frozen sample was vacuum-dried and then scanned under an electron microscope. Its micromorphology is shown in Figure 9.

Figure 9a illustrates the presence of a three-dimensional network structure in the gel system when observed at a microscopic level. Additionally, the molecular chains are densely packed together. Figure 9b demonstrates that the presence of polyethyleneimine strengthens the cross-linked structure, hence enhancing the microstructural strength of the gel system.

## 3. Conclusions

This study aimed to create a gel that can withstand high temperatures and high salt concentrations in order to effectively block water in reservoirs. The gel was developed using AM/AMPS as the polymer, polyethyleneimine as the cross-linking agent, and nylon fiber as the stabilizer. The optimal gel system was identified through dosage optimization tests, consisting of 1.0% AM/AMPS, 0.1% polyethyleneimine, and 0.5% nylon fiber.

Experiments were conducted to test the gel system’s resistance to temperature and salt, as well as its plugging performance. The findings from the temperature and salt resistance experiments indicate that the gel system’s syneresis rate stayed consistently below 2% after 120 days at a temperature of 130 °C. Similarly, in the salt resistance trials, the syneresis rate of the gel system remained below 2.5% after 120 days at 130 °C in a solution with a salt concentration of 230,720 mg/L. The findings of the plugging performance trials demonstrate that the gel system achieved a plugging rate of over 94% for sand-filled pipes with varying permeability, indicating a highly effective blocking effect.

A gel system with high temperature and high salt resistance has been constructed and assessed. However, this research primarily focused on the stability of the gel in a high-temperature and high salt-environment, and so the gel’s degradability has not been investigated. The gel’s degradability is a key criterion for determining its environmental friendliness. It will be considered when assessing the gel’s performance in future research.

## 4. Materials and Methods

### 4.1. Experimental Supplies

The polymer AM/AMPS utilized in this paper was made by aqueous solution polymerization; the mass fractions of AM and AMPS were 80% and 20%, respectively; acrylamide (AM); 2-acrylamido-2-methlpropanesulfonic acid (AMPS); polyethyleneimine (PEI); analytical; Shanghai Aladdin Biochemical Technology Co., Ltd., Shanghai, China; nylon fiber, sodium hydroxide, ammonium persulfate, and analytical purity, Shanghai Macklin Biochemical Technology Co., Ltd., Shanghai, China.

The simulated formation water utilized in this paper was prepared using the data provided by the oil field site, with a total salinity of 230,720 mg/L. A series of analytically pure chemicals, including NaCl, MgCl_2_, and CaCl_2_, utilized in the simulated formation water were purchased from Tianjin huhengda Chemical Co., Ltd., Tianjin, China.

### 4.2. Experimental Apparatus

The main apparatuses are shown in Table 3.

### 4.3. Experimental Method

#### 4.3.1. Preparation of the AM/AMPS Copolymer

**Figure 10:**
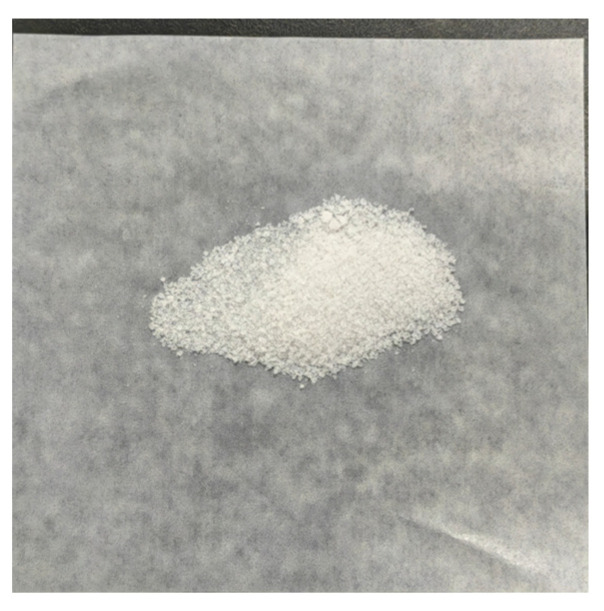
AM/AMPS copolymer particles.

AMPS and AM, with mass fractions of 20% and 80%, respectively, were combined at room temperature in a beaker. Distilled water, with a mass fraction of 80%, was added and stirred until both monomers were completely dissolved. The pH of the solution was modified to a range of 6 to 7 using a concentrated solution of sodium hydroxide. Subsequently, the solution was moved to a water bath maintained at a constant temperature of 40 °C. Once the temperature of the solution reached a steady state, ammonium persulfate was added, and then the reaction was maintained at a constant temperature for a duration of 12 h. Ultimately, the substance underwent purification using anhydrous ethanol and was thereafter dried at a temperature of 40 °C, resulting in the acquisition of white granular particles (Figure 10).

#### 4.3.2. Preparation Method of Temperature-Resistant and Salt-Resistant Gel

The gel system consisted of a polymer, a cross-linking agent, and a stabilizer. Initially, polyethyleneimine was added into the simulated formation water at room temperature based on the predetermined mass fraction, and agitated until entirely dissolved. Subsequently, nylon fiber with a mass fraction of 0.5% was introduced and agitated until it fully dissolved. A solution containing AM/AMPS with a mass fraction of 1.0% was added and simultaneously agitated. Subsequently, the fully dissolved homogeneous gel solution was transferred into an ampoule bottle and sealed. The ampoule bottle was then heated in an oven at a specific temperature to allow gelation to occur.

#### 4.3.3. Determination of Gel Syneresis Rate

This article evaluates the thermal stability of the gel through the syneresis rate [28]. The initial weight of the gel and the weight of water discharged from the gel after several days of aging were measured, and the syneresis rate was defined as the proportion of the weight of the water expelled after a period of gel aging to the original weight of the gel.

(1) $$R_{s}=\frac{m_{1}}{m_{0}}\times 100\%$$

In the equation: Rs is the syneresis rate of the gel, m_1_ is the weight of water expelled after the gel was aged for several days, and m_0_ is the original test weight of the gel.

#### 4.3.4. Determination of Gel Strength

This article used a visual method to determine the gel strength. The gel strength was determined by inverting the test tube and observing the gel state in the test tube. The strength classification standard adopted the Sydansk gel strength code [29,30], which is used in water blockage adjustment at home and abroad, as shown in Table 4. It is widely used in cross-sectional studies and is suitable for the determination of gel strength in test tubes.

#### 4.3.5. Blocking Performance Test

**Figure 11:**
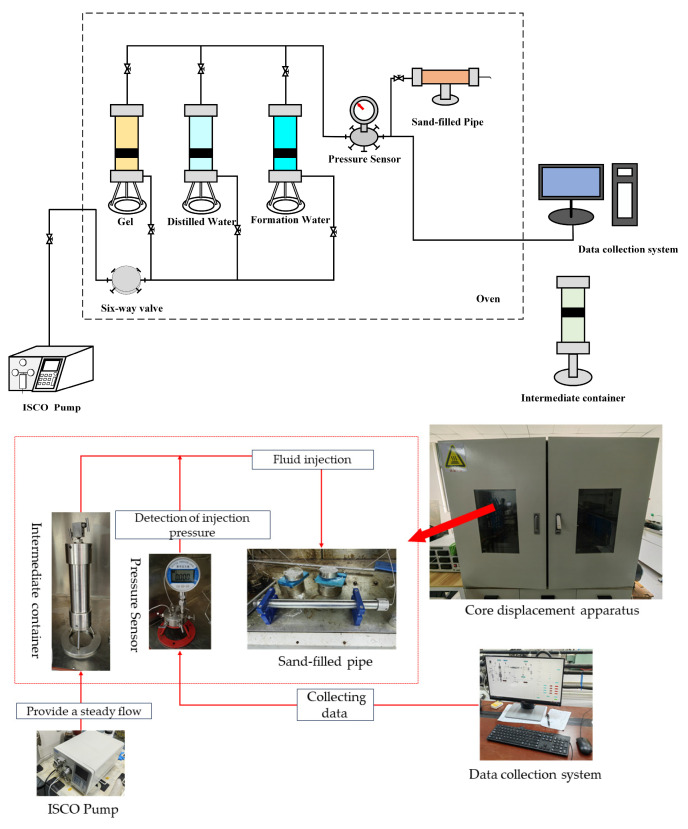
Single-tube experimental model.

This article used the plugging rate to characterize and evaluate the gel blocking performance. The blocking experiment used a single-tube experimental model (Figure 11).

Experimental steps:

1. Add the quartz sand into the sand-filled pipe, attach the filled pipe to the pressure sensor, activate the constant-flux pump (ISCO Pump), and remove any air from the attached lines leading to the intermediate container.

2. Set the injection rate to 0.5 mL/min and saturate the sand-filled pipe with distilled water until there is a consistent flow of water droplets from the outlet end of the pipe.

3. Measure the weight of the sand-filled pipe and record it as m_2_. Dry the sand-filled pipe, weigh it again, and record the weight as m_3_. Calculate the pore volume V_0_ (PV) (Equation (2)):

(2) $$V_{0}=\frac{m_{2}-m_{3}}{\rho_{0}}$$

In the equation: ρ_0_ is the density of distilled water.

4. Inject the simulated formation water into the sand-filled pipe, monitor the pressure sensor signals gathered in the data collection system, note the injection pressure, and compute the permeability k_0_ before blockage using Darcy’s law.

5. Inject 0.5 PV of gel-forming fluid into the sand-filled pipe at a rate of 0.5 mL/min.

6. Set the oven temperature, position the sand-filled pipe at the designated experimental temperature, and allow the gel-forming fluid to form a gel.

7. Once the fluid forms a gel, activate the ISCO Pump and inject the simulated formation water at a rate of 0.5 mL/min. Monitor the pressure sensor reading, note the injection pressure, and calculate the permeability of the plugging, denoted as k_1_. Determine the plugging rate E by utilizing the permeability values from the two calculations, as specified in Equation (3).

(3) $$E=\frac{k_{0}-k_{1}}{k_{0}}\times 100\%$$

In the equation: E is the plugging rate, %; k_0_ is the permeability before plugging, μm^2^; and k_1_ is the permeability after plugging, μm^2^.

## Figures and Tables

**Table 1:** Plugging test results.

No.	Permeability before Plugging/(10^−3^ μm^2^)	Permeability after Plugging/(10^−3^ μm^2^)	Breakthrough Pressure Gradient/(Mpa·m^−1^)	Plugging Rate/%
1	732.66	42.49	14.22	94.2
2	974.82	51.67	9.66	94.7
3	1307.32	61.44	8.04	95.3
4	1728.51	76.05	5.58	95.6
5	2543.36	94.10	4.88	96.3

**Table 2:** Physical parameters of sand-filled pipe.

Category	Quartz Sand (Mesh Size)	Length (cm)	Diameter (cm)
Hypertonic	120–200	50	2.5
Hypotonic	120–200	50	2.5

**Table 3:** The main apparatuses.

Apparatus Name	Type	Manufacturer
Precision Electronic balance	FA3004	Shanghai Sunny Hengping Scientific Instrument Co., Ltd., (Shanghai, China)
Constant-temperature water bath	DZTW	Jiangsu Unipac Technology Co., Ltd., (Haian, China)
Microscopic Visualization Body Device	U-FD-01	Jiangsu Unipac Technology Co., Ltd., (Haian, China)
Electric mixer	JJ-1	Beijing Ruili Analytical Instrument Co., Ltd., (Beijing, China)
Core displacement apparatus	UZCQ-50	Jiangsu Unipac Technology Co., Ltd., (Haian, China)
Constant-flux pump	2PB	Beijing Xingda Science & Technology Development Co., Ltd., (Beijing, China)
Digital Pressure Gauge	HCYS-100	Shanghai Ruyi Instrument Co., Ltd., (Shanghai, China)
Electro-Thermostatic Blast Oven	OHG-9073B5-III	Shanghai CIMO Medical Instrument Manufacturing Co., Ltd., (Shanghai, China)

**Table 4:** Gel Strength Code.

Gel Strength Code	Gel Description
A	No detectable gel formed: the gel appears to have the same viscosity as the original polymer solution
B	Highly flowing gel: the gel seems to be only slightly more viscous than the initial polymer solution
C	Flowing gel: most of the gel flows to the bottle cap by gravity upon inversion
D	Moderately flowing gel: only a tiny portion (5–10%) of the gel does not readily flow to the bottle cap by gravity upon inversion
E	Barely flowing gel: the gel can barely flow to the bottle cap, and a significant portion (>15%) of the gel does not flow by gravity upon inversion
F	Highly deformable non-flowing gel: the gel does not flow to the bottle cap by gravity upon inversion
G	Moderately deformable non-flowing gel: the gel deforms about halfway down the bottle by gravity upon inversion
H	Slightly deformable non-flowing gel: only the gel surface slightly bends by gravity upon inversion
I	Rigid gel: there is no gel surface deformation by gravity upon inversion

## Data Availability

The data presented in this study are openly available in the article.
